# Response to neoadjuvant chemotherapy based on pathologic complete response in very young patients with ER-positive breast cancer: a large, multicenter, observational study

**DOI:** 10.1186/s12885-021-08355-w

**Published:** 2021-05-31

**Authors:** Joohyun Woo, Se Jeong Oh, Jeong-Yoon Song, Byung Joo Chae, Jung Eun Choi, Jeeyeon Lee, Heung Kyu Park, Korean Breast Cancer Society, Woosung Lim

**Affiliations:** 1 Department of Surgery, Ewha Womans University Mokdong Hospital, Ewha Womans University College of Medicine, 1071, Anyangcheon-ro, Yancheon-gu, Seoul, 07985 Republic of Korea; 2 Department of Surgery, College of Medicine, The Catholic University of Korea, Incheon, Republic of Korea; 3 Department of Surgery, Kyung Hee University Hospital at Gangdong, Seoul, Republic of Korea; 4 Department of Surgery, Division of Breast Surgery, Samsung Medical Center, Sungkyunkwan University School of Medicine, Seoul, Republic of Korea; 5 Department of Surgery, Yeungnam University Hospital, Deagu, Republic of Korea; 6 Department of Surgery, School of Medicine, Kyungpook National University, Daegu, Republic of Korea; 7 Department of Breast Surgery, Gachon university Gil Medical Center, Incheon, Republic of Korea

**Keywords:** Breast cancer, Young patients, Neoadjuvant chemotherapy, ER-positive, Pathologic complete response

## Abstract

**Background:**

In estrogen receptor (ER)-positive breast cancer (BC), young age is associated with poor prognosis. While very young patients respond better to chemotherapy, chemotherapy is less effective in ER-positive tumors than in ER-negative tumors. The authors tried to evaluate chemotherapy response of very young patients with ER-positive BC by pathologic complete response (pCR) after neoadjuvant chemotherapy excluding the effect of endocrine treatment to the extent possible.

**Methods:**

We collected individual patient data from 1992 to 2013 from the Korean Breast Cancer Society (KBCS). Total 1048 ER-positive and 797 ER-negative patients aged < 50 years who had been treated with neoadjuvant chemotherapy were included for analysis. We compared pCR rate between patients aged < 35 years with ER-positive tumors and the other groups.

**Results:**

The proportion of patients aged < 35 years was 14.0% of patients with ER-positive BC in this cohort of under 50 years old, and 16.8% of patients with ER-negative BC in this cohort of under 50 years old. Although most characteristics of tumors according to age were comparable, tumors with high Ki-67 expression were more common in patients aged < 35 years than in patients aged 35-49 years in both ER-positive and -negative group (*P* = 0.001). Breast conservation rates were not significantly different according to age (44.2% vs. 46.8% in ER-positive group, 55.2% vs. 48.0% in ER-negative group). pCR rate was not different according to age in ER-positive group (*P* = 0.71) but significantly better in patients aged < 35 years in ER-negative group (*P* = 0.009). After adjusting for confounding variables, young patients maintained the higher probability of pCR than older patients in ER-negative tumors. However, pCR rate did not differ according to age in ER-positive tumors. In multivariate analysis, young age (< 35 years) was correlated with poor overall survival (*P* = 0.003, HR = 1.98) and there was only one event in a few patients achieved pCR in ER-positive group.

**Conclusions:**

Chemotherapy response based on pCR was not better in young patients (< 35 years) with ER-positive BC than in older premenopausal patients with non-metastatic ER-positive BC. Young age cannot be a predictive factor of response to neoadjuvant chemotherapy in ER-positive BC. Different biological characteristics such as high proliferative index should be considered.

**Trial registration:**

Retrospectively registered.

## Background

The incidence of breast cancer in very young women aged < 35 years is higher in Korea (~ 9.5%) than in Western countries (< 6%) [1, 2]. Young age at diagnosis is associated with poor prognosis. When compared with older women, young women are at a higher risk of having poorly differentiated and highly proliferative breast cancer [3]. Tumors in young women more often showed higher S-phase fractions and abnormal p53 expression [4]. In addition, these women were at higher risk of having more positive lymph nodes and larger tumors [5]. Young women are typically diagnosed with more advanced disease, which can be attributed to delayed diagnosis owing to the expected low incidence in young women, lower sensitivity of mammography in dense breasts, pregnancy, and lactation [6–8]. Young women are at a significantly increased risk of dying after adjustment for known prognostic factors and expected mortality [9].

Breast cancer is less ER-positive in younger women than in older women. It has been reported that the proportion of ER-positive tumor is 48–52% in young patients [3, 4, 10]. Survival outcomes are significantly worse in young patients than in older patients with ER-positive tumors. However, the prognosis did not differ between young patients and older patients with ER-negative tumors [11–13]. Outcome difference patterns between age groups were seen only in patients with ER-positive tumors. Several reports have suggested endocrine mechanisms as the possible underlying reason. Worse outcomes of young patients with ER positive tumors would be incompletely explained by a lower chance of chemotherapy-induced amenorrhea and the resistance to tamoxifen in adjuvant treatment in ER-positive tumor [11, 12, 14].

Conversely, it has been reported that adjuvant chemotherapy is less effective in ER-positive tumors than in ER-negative tumors. A trend towards an advantage for younger women under 35 years was observed [15]. In addition, the effect of age on response to chemotherapy according to the ER status has not been sufficiently assessed. Herein, the authors hypothesized that response to chemotherapy is a cause of poor prognosis in young women with ER-positive breast cancer and tried to evaluate the chemoresistance to and benefits from neoadjuvant chemotherapy in young patients with ER-positive breast cancer while excluding the effect of endocrine treatment as much as possible.

## Methods

### Patient population

**Fig. 1:**
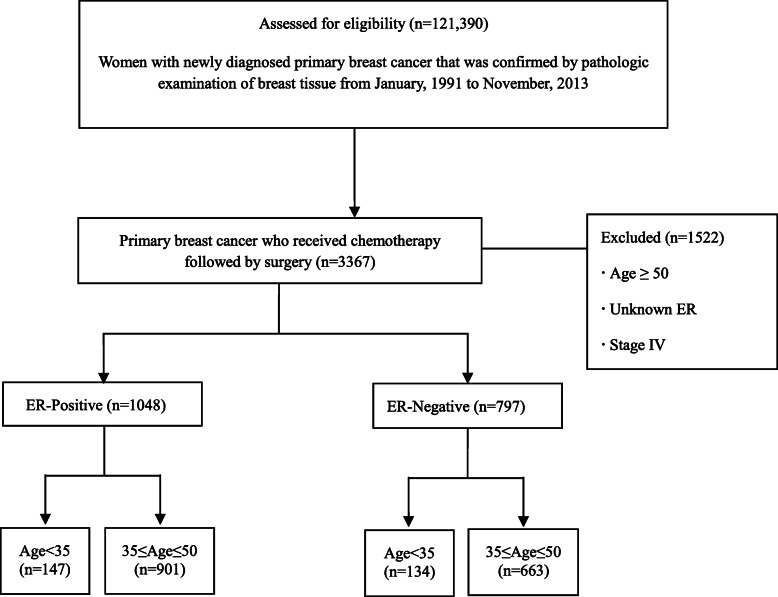
CONSORT diagram

In this study, all patients with primary breast cancer who received neoadjuvant chemotherapy followed by surgery between August, 1991 and November, 2013 were included for analysis. Patients with distant metastasis at diagnosis or unknown ER status and male patients were excluded (Fig. 1). This study was approved by the institutional review board of the Ewha Clinical Trial Center at Ewha Womans University Medical Center, Korea.

### Retrospective data collection

We collected individual patient data from the Korean Breast Cancer Society (KBCS). Korean Breast Cancer Registry (KBCR) is a collection of medical information relating to breast cancer patients. Nationwide, breast surgeons in a total of 102 general hospitals (41 university hospitals and 61 teaching hospitals) with 400 or more beds participate voluntarily in this registry since 1996 [14]. Individual hospitals recruited new patients who were diagnosed with breast cancer by pathologic examination of breast tissue and sent prospectively collected data of these patients to KBCS in the form of a filled-up recorded cancer registration sheet. In 2001, KBCS developed an online registration system, which made it more convenient for surgeons to enter patient enrollment data themselves. This web-based database has been managed by KBCS. The patients who were newly diagnosed as having breast cancer between 1992 and 1996, which is before the registration system was introduced, were recruited retrospectively. Data of these patients were collected from the database of each individual hospital and added to the KBCS database. This database comprises essential patient information, including unique registration number, the date of diagnosis and surgery, age at surgery, gender, the surgical method used, and clinical and pathologic tumor stage according the American Joint Committee on Cancer classification. It contains optional information, such as information on clinical and laboratory findings before surgery, tumor size, nodal status, histologic grade and type, Ki-67 proliferation index after surgery and before chemotherapy in case of neoadjuvant chemotherapy, and nuclear grade and biologic markers, including the expression status of estrogen receptor (ER), progesterone receptor (PR) and human epidermal growth factor receptor 2 (HER2). The goals of chemotherapy, such as neoadjuvant, adjuvant, or palliative therapy, and information about the administered chemotherapy regimen, radiotherapy, and endocrine therapy are recorded as treatment details. Even currently, these items are prospectively recorded starting from diagnosis of breast cancer, and overall survival data and the cause of death have been updated consistently through the Korean Central Cancer Registry, Ministry of Health and Welfare, Korea. Enrolled patients in this registry had given written informed consent for data collection at diagnosis. The database was reviewed to identify patients who met the criteria.

### Variables

Patients with no evidence of invasive residual tumor in breast or nodes irrespective of noninvasive components in the final surgical specimen were considered to have obtained pCR. Because not all cases could be confirmed histologically for lymph node metastasis before neoadjuvant chemotherapy was started, in these cases, total pCR and only-breast pCR statuses could be obtained by pathologic stage after surgery. Total pCR, not only-breast pCR was analyzed to identify the association of clinical factors or survival rate with chemotherapy response. The status of ER, PR, and HER2 was classified as negative or positive through immunohistochemistry (IHC) in individual hospitals. For the classification of the HER2 status, if the IHC result was a score of 2+, which was considered as equivocal, the result of FISH was reflected. TNM (tumor size, node status, and metastasis) stages determined before 2010 were re-classified according to the seventh edition of the TNM classification. The Ki-67 index was categorized into low and high according to the 2013 St. Gallen consensus [16]. If the Ki-67 index information was not available, histologic grade III was considered as highly proliferative tumor and histologic grades I or II were considered as low proliferative tumors. The chemotherapy regimen administered was classified as anthracycline-based or not. Younger patient group comprised patients below the age of 35 years and the older group comprised those aged 35–49 years.

### Statistical analysis

SPSS software version 20 (SPSS, Chicago, IL, USA) was used for all statistical analyses. Basic data on the characteristics of study subject groups were compared and analyzed using Chi-squared tests. The mean values of the subgroups were compared with each other using independent sample t-tests (Student t-test) to examine statistical significance. The association between clinicopathologic parameters and pCR rate within each subgroup was evaluated using the Chi-squared test and univariate Cox regression analysis. Furthermore, multivariate Cox regression analysis was used to determine the independent prognostic value within the stratified cohorts. Overall survival (OS) was defined as the time from the first diagnosis of breast cancer to death from any cause. Survival curves were assessed using the Kaplan-Meier method and comparison of survival curves was analyzed using the log-rank test. Multivariate analyses were conducted by using Cox’s proportional hazard regression model. A *p*-value of < 0.05 for a two-sided test was considered statistically significant.

## Results

### Clinicopathological characteristics of the study population

As authors expected, young patients aged < 35 years were much fewer than older patients aged 35–49 years in both ER-positive and -negative groups (14.0 and 16.8%). The proportion of young patients was comparable in the two groups (*P* = 0.099).

Most baseline characteristics were comparable irrespective of age in each group (Table 1). However, in the ER-negative group, there were significantly more HER2-negative tumors in young patients than in older patients (*P* = 0.012). In addition, the proportion of patients with high proliferative index was higher in young patients in both ER-positive and -negative groups (35.4 and 67.9%); however, the proportion of patients with high proliferative index was smaller than that with low proliferative index in the ER-positive group irrespective of age.

**Table 1:** Clinicopathological parameters

Variables	ER-Positive			ER-Negative		
	Age<35	35≤Age≤50	*P*-value	Age<35	35≤Age≤50	*P*-value
	(*N* = 147)	(*N* = 901)		(*N* = 134)	(*N* = 663)	
Year of diagnosis						0.585
1991–1995	1 (0.7)	3 (0.3)		1 (0.7)	2 (0.3)	
1996–2000	4 (2.7)	3 (0.3)		1 (0.7)	8 (1.2)	
2001–2005	24 (16.3)	111 (12.3)		25 (18.7)	97 (14.6)	
2006–2010	90 (61.2)	557 (61.8)		75 (56.0)	411 (62.5)	
2011–2013	28 (19.0)	227 (25.2)		32 (23.9)	145 (23.9)	
Histology			0.696			0.924
Ductal	140 (95.2)	846 (93.9)		128 (95.5)	633 (95.5)	
Lobular	3 (2.0)	30 (3.3)		0 (0.0)	2 (0.3)	
Other	1 (0.7)	5 (0.6)		2 (1.5)	8 (1.2)	
Unknown	3 (2.0)	20 (2.2)		4 (3.0)	20 (3.0)	
PR			0.379			0.674
Negative	45 (30.6)	244 (27.1)		115 (85.8)	578 (87.2)	
Positive	102 (69.4)	656 (72.8)		19 (14.2)	85 (12.8)	
Unknown	0 (0.0)	1 (0.1)		0 (0.0)	0 (0.0)	
HER2			0.404			0.012
Negative	106 (73.6)	674 (75.6)		104 (77.6)	429 (64.9)	
Positive	37 (25.7)	215 (24.1)		29 (21.6)	218 (33.0)	
Unknown	4 (2.7)	12 (1.3)		1 (0.7)	16 (2.4)	
Clinical Tumor size			1.000			0.745
≤ 2 cm	3 (2.0)	20 (2.2)		2 (1.5)	14 (2.1)	
> 2 cm	49 (33.3)	290 (32.2)		51 (38.1)	210 (31.7)	
Unknown	95 (64.6)	591 (65.6)		81 (60.4)	439 (66.2)	
Palpability			0.175			0.160
Nonpalpable	1 (0.7)	29 (3.2)		1 (0.7)	27 (4.1)	
Palpable	113 (76.9	650 (72.1)		95 (70.9)	459 (69.2)	
Unknown	33 (22.4)	222 (24.6)		38 (28.4)	177 (26.7)	
Nucleus grade			0.110			0.102
I	10 (6.8)	85 (9.4)		1 (0.7)	14 (2.1)	
II	48 (32.7)	359 (39.8)		28 (20.9)	106 (16.0)	
III	54 (36.7)	271 (30.1)		57 (42.6)	350 (52.8)	
Unknown	35 (23.8)	186 (20.6)		48 (35.8)	193 (29.1)	
Proliferative index			0.001			0.006
Low	79 (53.7)	612 (67.9)		19 (14.2)	184 (27.8)	
High	52 (35.4)	210 (23.3)		91 (67.9)	378 (57.0)	
Unknown	16 (10.9)	79 (8.8)		24 (17.9)	74 (11.2)	

NOTE: Data are presented as No. (%) unless otherwise specified

*ER* estrogen receptor, *PR* progesterone receptor, *HER2* human epidermal growth factor receptor 2

### Surgery and neoadjuvant chemotherapy

More than 70% of patients in both young and older patients received neoadjuvant chemotherapy with anthracycline-based regimen (Table 2). The proportion of patients who were administered concurrent trastuzumab as neoadjuvant treatment varied and were less than 9%. The proportion of HER2-positive patients treated with trastuzumab was comparable between young and older patients in both ER-positive and -negative groups (Table 2).

**Table 2:** Surgery and neoadjuvant chemotherapy

Characteristic	ER-Positive			ER-Negative		
	Age<35	35≤Age<50	*P*-value	Age<35	35≤Age<50	*P*-value
	(*N* = 147)	(*N* = 901)		(*N* = 134)	(*N* = 663)	
Breast surgery			0.626			0.125
Conserving	66 (44.9)	424 (47.1)		74 (55.2)	318 (48.0)	
Mastectomy	81 (55.1)	477 (52.9)		60 (44.8)	345 (52.0)	
Axillary surgery			0.561			0.005
SLNB	19 (16.4)	90 (10.0)		27 (20.1)	69 (10.4)	
SLNB➔ALND	43 (26.0)	269 (29.9)		27 (20.1)	174 (26.2)	
ALND	85 (56.2)	540 (59.9)		80 (59.7)	420 (63.3)	
Unknown	0 (0.0)	2 (0.3)		0 (0.0)	0 (0.0)	
Neoadjuvant chemotherapy			0.090			0.068
Anthracycline-based	111 (75.5)	689 (76.5)		107 (79.9)	504 (74.7)	
Trastuzumab-containg	13 (8.8)	515 (5.7)	0.138	5 (3.7)	58 (8.7)	0.053
Others	8 (5.4)	94 (10.4)		9 (6.7)	82 (12.1)	
Unknown	28 (19.0)	118 (13.1)		18 (13.4)	89 (13.2)	

NOTE: Data are presented as No. (%) unless otherwise specified

*ER* estrogen receptor, *SLNB* sentinel lymph node biopsy, *ALND* axillary lymph node dissection

With regard to surgical treatment, the rate of breast-conserving surgery was not different between young and older patients in both ER-positive and -negative groups. Although the axillary lymph node status by pathological confirmation before neoadjuvant chemotherapy was uncertain, approximately > 80% patients in all groups received axillary lymph node dissection.

### Association of clinical factors with pCR

In univariate analysis, pCR was achieved in young patients with nonpalpable tumors. It was also achieved in patients with ER- and PR-negative tumors and high proliferative index. Odds ratio of ER status was higher than that of age. When patients were stratified by their ER status, independent prognostic factors of pCR changed in young and older patients. Negative PR and high proliferative index were positive factors for pCR in the ER-positive group, and age < 35 years was the only one in the ER-negative group. Patients with nonpalpable tumors had a higher tendency to achieve pCR in both groups (Table 3).

**Table 3:** Univariate and multivariate analysis for pathologic complete response

Variables	ER-Positive (*N* = 1048)				ER-Negative (*N* = 797)			
	Univariate		Multivariate		Univariate		Multivariate	
	OR (95% CI)	*P*-value	OR (95% CI)	*P*-value	OR (95% CI)	*P*-value	OR (95% CI)	*P*-value
Age<35 vs. 35≤Age<50	1.17 (0.51–2.67)	0.71	0.41 (0.05–3.19)	0.39	1.92 (1.17–3.13)	0.01	2.54 (1.27–5.08)	0.01
PR-negative vs. –positive	1.87 (1.01–3.47)	0.05	2.00 (0.68–5.88)	0.21	1.60 (0.78–3.27)	0.20	5.45 (1.26–23.61)	0.02
HER2-positive vs. –negative	0.67 (0.33–1.37)	0.28	NI		1.33 (0.82–2.16)	0.25	NI	
Proliferative index: high vs. low	2.30 (1.02–5.20)	0.05	3.45 (1.17–10.15)	0.03	1.00 (0.58–1.74)	0.99	1.22 (0.60–2.46)	0.58
Nonpalpable vs. palpable	3.28 (0.93–11.54)	0.06	1.61 (0.20–13.16)	0.66	2.27 (0.93–5.53)	0.07	3.95 (1.32–11.8)	0.01

*ER* estrogen receptor, *PR* progesterone receptor, *HER2* human epidermal growth factor receptor 2, *OR* odds ratio, *CI* confidence interval, *NI* not included in the model

For multivariate analysis, the model was constructed on the basis of meaningful variables from univariate analysis, potential confounding variables in both ER-positive and -negative groups, and palpability of the tumor. Factors affecting pCR differed according to the ER status (Table 3).

The pCR rate of young patients was higher than that of older patients in the ER-negative group but was comparable in the ER-positive group (Table 4). Even after adjusting for confounding variables, young patients maintained the higher probability of pCR than older patients in ER-negative tumors. However, the pCR rate did not differ according to age in ER-positive tumors. In addition, the odds of pCR rate changed from greater than 1 into less than 1 after adjustment (Table 5).

**Table 4:** Pathologic results after neoadjuvant chemotherapy and response to chemotherapy

Characteristic	ER-Positive			ER-Negative		
	Age<35	35≤Age<50	*P*-value	Age<35	35≤Age<50	*P*-value
	(*N* = 147)	(*N* = 901)		(*N* = 134)	(*N* = 663)	
Postoperative T stage						
ypT0, ypTis	6 (4.1)	27 (3.0)	0.638	28 (20.9)	111 (16.8)	0.310
ypT1-ypT4	141 (95.9)	874 (97.0)		106 (79.1)	552 (83.2)	
ypT1	47 (32.2)	351 (39.1)		29 (21.6)	188 (28.4)	
ypT2	69 (47.3)	348 (38.8)		45 (33.6)	204 (30.8)	
ypT3	22 (15.1)	158 (17.6)		23 (17.2)	94 (14.2)	
ypT4	2 (1.4)	14 (1.3)		0 (0)	0 (0)	
Postoperative N stage						
ypN0	55 (37.4)	296 (32.9)	0.515	77 (57.5)	301 (45.4)	0.016
pN1-ypN3	92 (62.6)	604 (67.0)		52 (38.8)	323 (47.9)	
Pathologic response						
Complete: breast and axilla	7 (4.8)	37 (4.1)	0.713	26 (19.4)	74 (11.2)	0.009
Complete: breast-only	1 (0.7)	3 (0.3)	1.000	3 (2.2)	45 (6.7)	0.006

NOTE: Data are presented as No. (%) unless otherwise specified

*ER* estrogen receptor

**Table 5:** Change of odds ratio after adjustment according to ER status

	pCR rate (%)		OR (95% CI)	PI-Adjusted OR (95% CI)
	Age<35	35≤Age<50		
ER-positive	(*N* = 147)	(*N* = 901)		
	4.8	4.1	1.17 (0.51–2.67)	0.41 (0.05–3.19)
ER-negative	(*N* = 134)	(*N* = 663)		
	19.4	11.2	1.92 (1.17–3.13)	2.54 (1.27–5.08)

*ER* estrogen receptor, *pCR* pathologic complete response, *OR* odds ratio, *CI* confidence interval, *PI* proliferative index

### Survival analysis

**Fig. 2:**
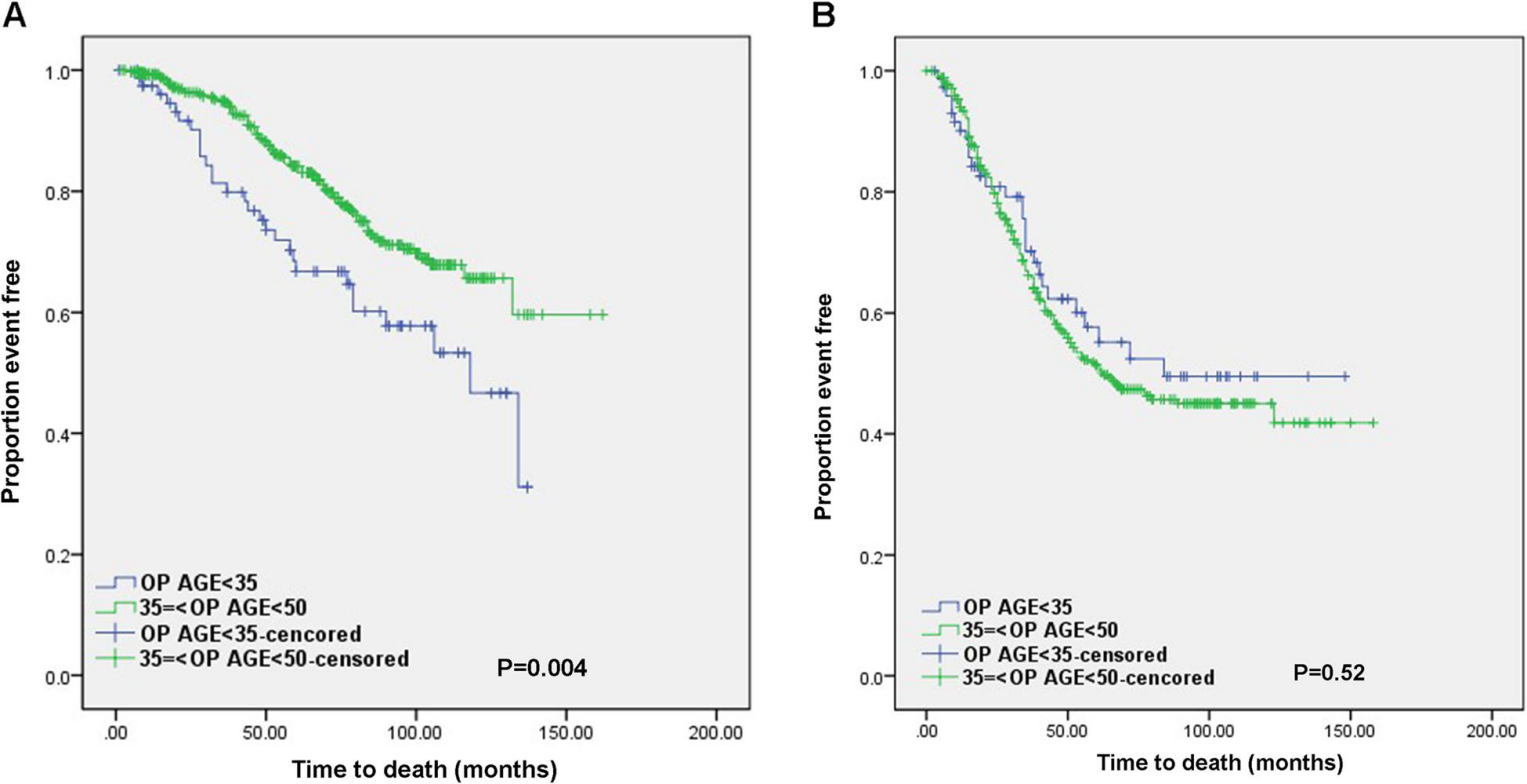
Overall survival according to age in **a** ER-positive or **b** ER-negative groups

**Fig. 3:**
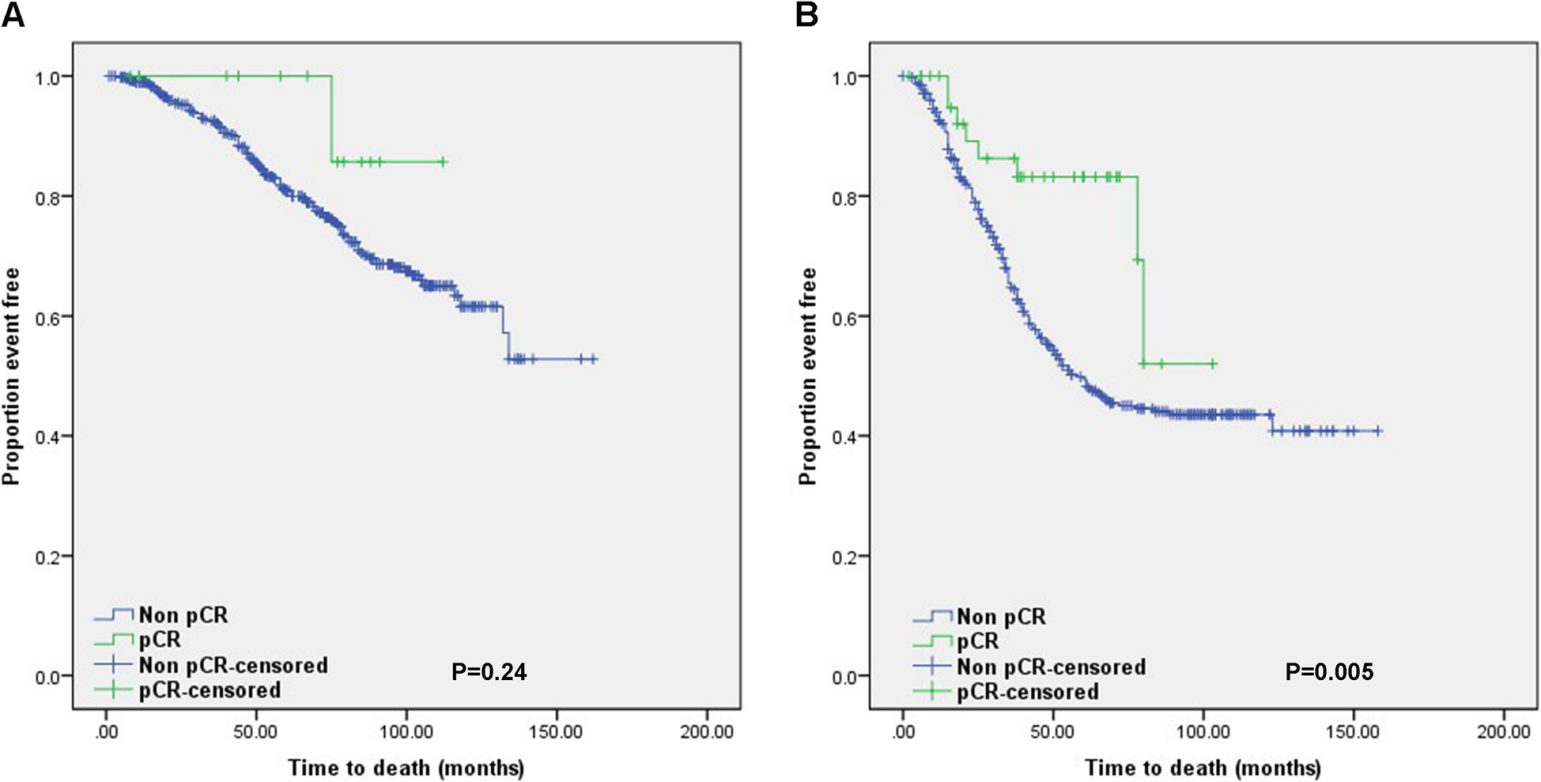
Overall survival according to pCR in **a** ER-positive or **b** ER-negative groups

The median follow-up time was 55 months. There have been 302 deaths (32.5%). One hundred sixteen patients (22.9%) died in ER-positive group and 186 patients (44.1%) died in ER-negative group. In univariate analysis, young patient aged under 35 of ER-positive group showed a significant poor OS compared to older patients. There was no significant difference in OS between young and older patients in ER-negative group (Fig. 2). pCR was correlated with a better OS in ER-negative group (Fig. 3) and subgroup analysis stratified by age showed similar relationship between pCR and OS in this group. Correlation of pCR with OS could not be clearly defined in ER-positive group because there was only one event in a few patients achieved pCR in this group. Age (age < 35 vs. 35 ≤ age < 50), pCR status (non-pCR vs. pCR), HER2 status (positive vs. negative) and proliferative index (high vs. low) were included as variables in Cox’s regression model for multivariate proportional hazards analysis. In multivariate analysis, young age under 35 years and high proliferative index were correlated with a poor overall survival in ER-poritive group (*P* = 0.003, 0.001, HR = 1.98, 1.70). Only non-pCR was associated with a poor overall survival in ER-negative group (*P* = 0.04, HR = 2.19). There was no relationship between pCR and overall survival in ER-positive groups in Log rank test and multivariate proportional hazards analysis (*P* = 0.239, 0.97). However, authors could not conclude that a significance did not exist because only two events had occurred in ER-positive group during the period of observation.

## Discussion

This large retrospective study showed the response to neoadjuvant chemotherapy of young patients aged < 35 years having ER-positive breast cancer. When other confounding factors were controlled, young patients were not found to respond to chemotherapy better than older patients in the ER-positive group. Although there was no change in odds ratio in the ER-negative group after adjustment, it was notable that odds ratio changed in the ER-positive group, albeit not statistically significantly. In other words, there was no difference of response to neoadjuvant chemotherapy beween young and older patients in ER-positive group while response to neoadjuvant chemotherapy of young patients was better than that of older pateints in ER-negative group.

Neoadjuvant chemotherapy is generally performed in patients with locally advanced breast cancers. Physicians tend to give patients with advanced disease more aggressive treatment because of their high tumor burden and increased risk of recurrence and death [17]. In addition, as chemotherapy is considered more effective in younger patients with poor prognosis and shorter disease-free survival regardless of the type of treatment, tumor size, grade or progesterone receptor status, these patients are administered neoadjuvant chemotherapy for breast-conserving surgery or a lower threshold when determining chemotherapy [1, 2, 18]. A large study of patients aged < 50 years reported that among patients with lower risk disease, those who were not treated with adjuvant systemic therapy and were aged < 35 years had the worst prognosis. This age-related trend was not seen in high-risk patients with a greater number of metastatic nodes or a larger primary tumor size. They suggested that young women with breast cancer should be regarded as high risk patients and be given adjuvant cytotoxic treatment on the basis of age alone [9]. This recommendation is under the assumption that young patients would respond better to cytotoxic treatment even in lower risk disease [19]. However, steroid hormone status was not considered in this analysis [9].

The association of young age with response to chemotherapy has not been clearly defined, particularly according to ER status [20]. In this study, young age was not an independent predictor of response to chemotherapy in ER-positive tumors. Patient age of < 35 years was found to be associated with pCR in ER-negative tumors but not in ER-positive tumors. Better response to chemotherapy was related to aggressiveness of tumors in ER-positive breast cancers. High proliferative index was an independent factor only in ER-positive tumors. Therefore, in ER-positive tumors, it is hard to expect that complete pathologic response would be achieved in low proliferative tumors, even though they developed in young patients. Conversely, Early Breast Cancer Trialists’ Collaborative Group reported that adding adjuvant polychemotherapy to tamoxifen was associated with 7.6% of 5-year survival gain in patients aged < 50 years with ER-positive tumors [21]. The survival benefit of chemotherapy in young patients was greater than in older patients. However, because survival benefit may not come from a cytotoxic effect of chemotherapy and the criteria for age were different, response to polychemotherapy in young women cannot be interpreted clearly. Furthermore as tamoxifen is the only endocrine therapy, the endocrine effect of chemotherapy would play an important role in benefit of chemotherapy for patients less than 50 years with ER-positive tumors as in most other studies.

The evidence of the benefit of adding chemotherapy to endocrine therapy in hormone-responsive breast cancer of young women is not strong [22, 23]. A pooled analysis of patients aged ≤40 years enrolled in EORTC trials showed that prolonged adjuvant CMF chemotherapy did not bring survival advantage to hormone receptor-positive patients compared with hormone receptor-negative patients [23]. Several studies suggest very good prognosis with optimal endocrine therapy alone, particularly in low-risk young patients [24]. Treatment advances with endocrine agents, such as ovarian function suppression or aromatase inhibitor, to overcome resistance to endocrine therapy in young breast cancer patients could improve patient outcomes without chemotherapy in young women with ER-positive breast cancers [25, 26]. Improvement of outcome in these patients is attributed to the endocrine effect. Particularly in ER-positive early breast cancer, the role of chemotherapy can be limited to endocrine effect, not cytotoxic effect. A STEPP analysis for patients with ER-positive tumors who received dose-intensive epirubicin and cyclophosphamide suggested a correlation between the achievement of ovarian function suppression and efficacy of chemotherapy despite no interaction of age and chemotherapy [27].

The bigger reason for the less benefit in these patients may be that ER-positive breast tumors resist chemotherapy. This study showed that the effect of ER status was greater than the effect of age. Some reports explain that the lower frequency of receptor positivity in premenopausal women may account for their increased response to cytotoxic chemotherapy [28]. Low response to chemotherapy of ER-positive breast cancers can counteract the effect of age. Even in palliative setting, endocrine treatment is recommended first in ER-positive breast cancer patients without visceral crisis. In addition, resistance to chemotherapy of estrogen receptor-positive breast tumors can be explained by biologic factors. Cancer stem cells in the ER-positive cells escape the effect of doxorubicin treatment by the elevation of p53 expression [29]. More tumors in young women had abnormal expression of p53 [4].

Predictive value for response to chemotherapy of young age in ER-positive breast cancers should be considered differently from that in ER-negative breast cancers. Most studies have focused on the endocrine effect of chemotherapy regarding the prognosis of young patients with ER-positive tumors. Although indirectly, this study shows the cytotoxic effect of chemotherapy in young patients with ER-positive tumors while excluding the endocrine effects as much as possible. Age was an important predictor of pCR in ER-negative tumors but not in ER-positive tumors in this study. In previous studies, it has been reported that young breast cancer patients had better response to chemotherapy than older breast cancer patients [30]. However, this result does not take ER status into consideration even though the proportion of ER-positive tumors was significantly different between both groups. A recent German study by Loibl et al. also reported that pCR was significantly higher following neoadjuvant treatment in patients aged < 35 years than in those aged over 35 years, and the outcomes were similar only for the triple-negative breast cancer subtype. The conventional perception that young women respond better to chemotherapy needs to be accepted as limited to ER-negative breast cancers. Even though only a small proportion of breast cancer patients in Korea carry BRCA mutations, BRCA mutation carriers have been reported in 15% of young Korean women with breast cancer. Also BRCA mutation carriers’ tumors are typically ER-negative. In very young women with breast cancers, the occurrence of early-onset breast cancer can be associated with BRCA mutations [31].

In ER-positive tumors, the effect of endocrine therapy following adjuvant chemotherapy makes it difficult to clearly determine the cytotoxic effect of chemotherapy only on the outcome in an adjuvant setting. Conversely, in a neoadjuvant setting, low rate of complete response to neoadjuvant chemotherapy may suggest resistance to chemotherapy because neoadjuvant chemotherapy is an in vivo measure of response to chemotherapy [32]. Although chemotherapy inevitably has an endocrine effect, endocrine effect o fhcemotherapy can be minimized in neoadjuvant setting.

This study showed that pCR rate of patients under 50 years in ER-positive group was low and pCR was not correlated with OS in ER-positive group. Response to chemotherapy based on pCR can have less impact on prognosis in ER-positive patients as pCR is a surrogate endpoint for improved OS mainly in aggressive tumor subtypes [33]. There are several reasons for this weak relationship between pCR and outcome of ER-positive tumors. Pathologic complete response means the effect of a chemotherapy only on the primary tumor, but does not mean the effect on micrometastases. Cancer is the potential micrometastatic systemic disease and residual cells may not respond to therapies that are effective against the primary tumor. Endocrine treatment for ER-positive tumors may work by other mechanisms not measured by the effect on the primary tumor even if it cannot lead to pCR [34].

In this study, there are some limitations because it is a retrospective cohort study using multicenter database. Even though TNM stage at diagnosis was recorded, the exact tumor size or axillary lymph node status before neoadjuvant chemotherapy was uncertain. Some patients did not receive fine needle aspiration or sentinel lymph node biopsy for axillary lymph nodes. Therefore, only patients with pathologic complete response were included in this study. In addition, the neoadjuvant chemotherapy regimen including trastuzumab administration was not the standard treatment during the study period. For HER2-positive tumors, this study does not reflect the current treatment. The POSH study demonstrated that long-term outcome over 8 years of young patients with ER-positive tumors was equally poor when compared to that of young patients with ER-negative tumors [35]. Longer follow-up time is needed to observe changes in longer-term survival.

In this study, young age under 35 years was not a predictor of pCR but the most important predictor of poor OS in ER-positive group. Analysis of 9885 breast cancer patients aged ≤50 years patients also showed risk of death from breast cancer increased abruptly in patients age under 35 years with hormone receptor-positive tumors [11]. Despite having limitations, this study suggests that young age alone is not an immediate factor in determining a chemotherapy escalation or neoadjuvant chemotherapy in ER-positive breast cancers. If other predictive factors for response to chemotherapy such as high proliferation index are not found, even increasing breast-conserving rate after neoadjuvant chemotherapy may be hard to expect.

## Conclusions

Chemotherapy response based on pCR rates was not better in young patients (< 35 years) than in older patients (35 ≤ age < 50) with ER-positive breast cancer. Response to chemotherapy may be predicted considering a different biology of young women such as high Ki-67 proliferative index in young patients with non-metastatic ER-positive tumors.

## Data Availability

The datasets during and/or analysed during the current study are available from the corresponding author on reasonable request.

## Abbreviations

ER: Estrogen receptor
HER2: Human epidermal growth factor receptor
IHC: Immunohistochemistry
KBCS: Korean Breast Cancer Society
OS: Overall survival
pCR: Pathologic complete response
PR: Progesterone receptor
TNM: Tumor size, node status, and metastasis
